# Chemokine Levels among Patients with Middle East Respiratory Syndrome Coronavirus Infection

**DOI:** 10.3390/vaccines11061048

**Published:** 2023-05-31

**Authors:** Abdulkarim Alhetheel, Ahmed Albarrag, Zahid Shakoor, Ali Somily, Mazin Barry, Haifa Altalhi, Muhammed Bakhrebah, Majed Nassar, Mohamed Alfageeh, Ayed Assiri, Sarah Alfaraj, Ziad Memish

**Affiliations:** 1 King Saud University Medical City, Riyadh 11362, Saudi Arabia; 2 Department of Pathology, College of Medicine, King Saud University, Riyadh 11451, Saudi Arabia; 3 Department of Infectious Diseases, College of Medicine, King Saud University, Riyadh 11362, Saudi Arabia; 4 Division of Infectious Diseases, Faculty of Medicine, University of Ottawa, Ottawa, ON K1H 8M5, Canada; 5 Advanced Diagnostics and Therapeutics Institute, Health Sector, King Abdulaziz City for Science and Technology, Riyadh 11442, Saudi Arabia; 6 Critical Care Unit, Prince Mohammed Bin Abdulaziz Hospital, Ministry of Health, Riyadh 11553, Saudi Arabia; 7 Corona Center, Prince Mohammed Bin Abdulaziz Hospital, Ministry of Health, Riyadh 11553, Saudi Arabia; dr.sara.fj@gmail.com; 8 Research and Innovation Center, King Saud Medical City, Ministry of Health, Riyadh 11553, Saudi Arabia; 9 College of Medicine, Alfaisal University, Riyadh 11553, Saudi Arabia

**Keywords:** chemokines, MERS-CoV, Saudi Arabia

## Abstract

Middle East respiratory syndrome coronavirus (MERS-CoV) is associated with significant morbidity and mortality due to intense pulmonary inflammation. Enhanced chemokine-mediated leukocyte infiltration in lungs has been linked with unfavorable outcomes with respect to the disease. This cross-sectional study assessed the levels of chemokines among 46 MERS-CoV-infected patients (19 asymptomatic and 27 symptomatic) and 52 healthy controls using a customized Luminex human chemokine magnetic multiplex panel. The plasma levels of interferon-inducible protein (IP)-10 (568.5 ± 114.7 vs. 55.19 ± 5.85 pg/mL; *p* < 0.0001), macrophage inflammatory protein (MIP)-1 alpha (MIP-1A) (30.78 ± 2.81 vs. 18.16 ± 0.91 pg/mL; *p* < 0.0001), MIP-1B (36.63 ± 4.25 vs. 25.26 ± 1.51 pg/mL; *p* < 0.003), monocyte chemoattractant protein (MCP)-1 (1267 ± 309.5 vs. 390.0 ± 35.51 pg/mL; *p* < 0.0002), and monokine-induced gamma interferon (MIG) (28.96 ± 3.93 vs. 16.29 ± 1.69 pg/mL; *p* < 0.001), interleukin (IL)-8 (147.9 ± 21.57 vs. 84.63 ± 10.62 pg/mL; *p* < 0.004) were significantly higher in symptomatic patients than healthy controls. Likewise, the levels of IP-10 (247.6 ± 80.09 vs. 55.19 ± 5.85 pg/mL; *p* < 0.0002) and MCP-1 (650.7 ± 149 pg/mL vs. 390 ± 35.51 pg/mL; *p* < 0.02) were also significantly higher in asymptomatic patients compared to healthy controls. However, no differences were observed in the plasma levels of MIP-1A, MIP-1B, MIG, and IL-8 between asymptomatic patients and uninfected controls. Conversely, the mean plasma levels of regulated on activation normal T cell expressed and secreted (RANTES) (3039 ± 301.0 vs. 4390 ± 223 pg/mL; *p* < 0.001) and eotaxin (176.9 ± 30.20 vs. 296.2 ± 28.11 pg/mL; *p* < 0.01) were significantly lower in symptomatic MERS-CoV-infected patients compared to healthy controls. Likewise, the levels of eotaxin (162.7 ± 21.60 vs. 296.2 ± 28.11 pg/mL; *p* < 0.01) were also significantly lower in asymptomatic patients. Interestingly, the level of MCP-1 (2139 ± 548.2 vs. 776.5 ± 165.3 pg/mL; *p* < 0.004) was significantly higher in deceased symptomatic patients compared to recovered symptomatic patients. MCP-1 was the only chemokine associated with a higher risk of mortality. Symptomatic MERS-CoV-infected patients had a significant elevation of plasma chemokines and elevated MCP-1 levels were found to be associated with fatal outcomes.

## 1. Introduction

The Middle East respiratory syndrome coronavirus (MERS-CoV) that first emerged in the Middle East in 2012 belongs to a group of beta-coronaviruses that also includes severe acute respiratory syndrome (SARS) coronavirus 1 and 2. This virus is capable of inflicting intense respiratory inflammation and is associated with substantial mortality, specifically among patients with comorbidities, such as chronic lung, heart, and kidney disorders, diabetes, or hypertension. MERS-CoV is a zoonotic virus considered to have originated from bats, with dromedary camels as intermediate hosts [1]. The mode of animal to human and human to human transmission of MERS-CoV remains unclear. Outbreaks of MERS-CoV infection have been reported in 27 different locations in the Arabian Peninsula since 2012. The clinical course of patients infected with MERS-CoV is variable, ranging from asymptomatic presentation or mild symptoms to intense disease and fatal outcome due to multiorgan failure [1]. Compared to other human coronaviruses, MERS-CoV exhibits the highest mortality rate of over 35%. Between 2012 to 2022 about 2600 confirmed infections and 935 deaths have been reported worldwide [2]. Both innate and adaptive immune responses are believed to play important roles in the pathogenesis of MERS-CoV infection; however, the exact mechanisms involved remain unclear [3]. Among the various immunological responses generated against MERS-CoV infection, production of proinflammatory cytokines and chemokines is considered to be the most critical immune response influencing the outcome of infection [3].

Chemokines are a family of small secreted proteins with molecular weight of about 7–15 kDa that play a critical role in immune cell trafficking and are involved in several physiological and pathological signaling activities [4,5]. These chemoattractant proteins are classified into four major groups based on the arrangement of their N-terminal cysteine residues, which determines their biological function and receptor specificity. The first group is CXC chemokines, which are also known as alpha-chemokines; these have one amino acid between the first two cysteine residues such as CXCL8, CXCL9, and CXCL10. They are involved in the recruitment of neutrophils, T cells, and natural killer cells. The second group is CC chemokines or beta-chemokines with adjacent cysteine residues such as CCL2, CCL5, and CCL21. They are involved in the recruitment of monocytes, eosinophils, basophils, and T cells. The third group is C chemokines, also known as gamma-chemokines, that have only two cysteine residues like XCL1 and XCL2 and they are involved in the recruitment of lymphocytes and dendritic cells. The fourth group is CX3C chemokines or delta-chemokines that have three amino acids between the first two cysteine residues, and contain a membrane-spanning domain which allows them to be expressed as both soluble and membrane-bound forms. The only known CX3C chemokine is CX3CL1 and it is involved in the recruitment of leukocytes. In addition to these main groups, there are also atypical chemokines such as CXCL16, which has a transmembrane domain and is involved in the recruitment of natural killer cells and T cells, and CXCL14, which lacks a disulfide bond and has a special receptor binding site and is involved in the recruitment of macrophages [4,5].

Chemokines are normally produced by activated macrophages, fibroblasts, epithelial, and endothelial cells to regulate the trafficking of white blood cells to the sites of inflammation, where they serve as the first line of defense against microbial infections [6,7]. Alterations in the induction or secretion of chemokines lead to dysregulation of immune tolerance and cell-mediated immune responses [8,9]. Development of MERS-CoV-related symptoms and complications has been linked with MERS-CoV infection and replication inside macrophages with expression of proinflammatory mediators [10,11]. These mediators include proinflammatory cytokines and chemokines such as interleukin (IL)-1β, IL-6, and IL-8, which is known as C-X-C motif chemokine ligand (CXCL)-8 [12]. Similarly, MERS-CoV infection of monocyte-derived macrophages and dendritic cells has been shown to release significantly higher concentrations of IL-2, IL-3, C-C motif chemokine ligand (CCL)-2, CCL3, and regulated on activation normal T cell expressed and secreted (RANTES), also known as CCL5 [12], IP-10 known as CXCL10, IL-12, and interferon (IFN)-γ [13]. MERS-CoV-infected macrophages and dendritic cells secrete higher concentration of IP-10, CCL2, CCL3, RANTES, and IL-8 chemokines, resulting in an influx of monocytes and macrophages in the infected tissues [13] and inducing tissue damage in the lower parts of the respiratory tracts [11].

Higher concentrations of macrophage and neutrophils attracting chemokines have been reported in bronchoalveolar lavage samples from COVID-19 patients. These chemokines are considered to perform a critical role in the induction of pulmonary dysfunction and pneumonia in COVID-19 by recruiting leukocytes in the lungs [14]. Higher concentrations of CXCL16 have been linked with moderate illness in COVID-19-infected patients [15]. Moreover, treatment of peripheral blood monocytes has been shown to increase the production of IP-10, CXCL11, CCL15, CCL16, and CCL19 chemokines [16]. In contrast, chemokines such as CCL2 [17] and IP-10 [18] have been shown to suppress the growth of hematopoietic stem cells in SARS-CoV infection resulting in frequently observed lymphopenia in the disorder [19]. Both chemokines CCL2 and IP-10 have also been implicated in lymphopenia characteristically observed among patients with COVID-19 [20]. In the backdrop of these observations, this study was performed to assess the chemokine profile (IP-10, MIG, MCP-1, MIP-1A, MIP-1B, IL-8, RANTES, eotaxin) in the peripheral blood of patients infected with MERS-CoV.

## 2. Subjects and Methods

This cross-sectional study was performed to assess the levels of chemokines among MERS-CoV patients between January 2018 and September 2019 at King Saud University Medical City, Riyadh. Forty-six MERS-CoV-infected patients (male = 22; female = 24) and a group of 52 healthy individuals (male = 51; female = 1) were enrolled (Table 1). Blood samples were collected from MERS-CoV patients at presentation. For each participant, whole blood was collected in a 5 mL vacutainer purple top tube containing ethylenediaminetetraacetic acid (EDTA) anticoagulant. The blood was then centrifuged at 35,000 rpm for 5 min (min) to separate the plasma layer. The plasma was collected in a 2 mL sterile cryotube and stored at −20 °C for future use. The diagnosis of MERS-CoV infection was confirmed via detection of both genes of MERS-CoV *upstream E* (*UpE*) and *open reading frame 1 a* (*Orf1a*) in nasopharyngeal swab sample in viral transport medium using real-time reverse transcription–polymerase chain reaction (RT-PCR). None of the patients was receiving any antiviral or immunosuppressive medications at the time of collection of the blood sample. All healthy individuals tested negative for hepatitis B virus, hepatitis C virus, human immunodeficiency virus (HIV), and human T-Cell lymphotropic virus (HTLV). These tests were performed using the Architect i2000 analyzer (Abbott, Chicago, IL, USA) and chemiluminescent microparticle immunoassay (CMIA) kits approved by the Food and Drug Administration (FDA). The cut-off value for the HBsAg kit was = 1 RLU (relative light unit)/mL; HCV Ab kit was = 1 RLU/mL; HIV Ag/Ab kit was = 1 RLU/mL; HTLV Ab kit was =1 RLU/mL. The study protocol was approved by the College of Medicine Institutional Review Board (IRB), King Saud University, (project #E15-1625). All participants signed an informed consent.

### 2.1. Measurement of Chemokine Levels Using Luminex Human Chemokine Magnetic Multiplex Panel

The plasma levels of the IP-10, MIG, MCP-1, MIP-1A, MIP-1B, IL-8, RANTES, and eotaxin chemokines were measured in patients and controls using a customized Luminex human chemokine magnetic multiplex panel (Novex Life Technologies, Carlsbad, CA, USA) according to the manufacturer’s instructions. Briefly, the standard of each human chemokine was prepared by adding 500 μL assay diluent in the lyophilized form of each chemokine and incubated at 25 °C for 15 min. Subsequently, 300 μL was transferred into a sterile tube (top standard) followed by preparation of 1:3 serial dilutions. Twenty-five microliters of antibody bead solution was then dispensed into a 96-well microtiter plate and incubated for 1 min on a magnetic stirrer. The plate was washed twice with 200 μL washing buffer followed by the addition of 50 μL incubation buffer. Subsequently, 50 μL of plasma sample combined with 50 μL assay diluent was added to each sample well, whereas 100 μL of standard was added to the standard wells. The plate was then covered and incubated at 4 °C overnight on a shaker in the dark. After incubation, each well was washed twice with 200 μL washing buffer followed by the addition of 100 μL biotinylated detector antibody and incubated for 1 h on a shaker in the dark. Each well on the plate was then washed twice with 200 μL washing buffer followed by the addition of 100 μL streptavidin R-Phycoerythrin (RPE) solution and incubated for 30 min on a shaker in the dark. Finally, each well was washed three times with 200 μL washing buffer followed by the addition of 150 μL washing buffer and the plate was shaken for 3 min before the processing of the plate using the Luminex MAGPIX system. The results were interpreted using the xPONENT software, with the concentrations of chemokines being expressed in pg/mL. The analytical sensitivities for MIG, MCP-1, MIP-1A, MIP-1B, and RANTES were <5 pg/mL; for IP-10 and eotaxin, they were <0.5 pg/mL; IL-8 was <1 pg/mL.

### 2.2. Diagnosis of MERS-CoV Infection Using a Qualitative Real-Time Reverse Transcription-Polymerase Chain Reaction (RT-PCR)

Extraction and detection of MERS-CoV RNA were performed as previously described [21]. Briefly, extraction of total nucleic acid from nasopharyngeal swab in viral transport medium aliquots was performed using the Nucleic Acid Isolation Kit I with the MagNA Pure Compact system (Roche Applied Science). Three hundred microliters of specimen was used for each extraction, with RNA eluted in a final volume of 50 µL. Subsequently, the master mix which contained a mixture of dNTPs, Taq polymerase, reverse transcriptase, primers, and probes for each target (*UpE* or *Orf1a*) was prepared according to the instructions of the RealStar^®^ MERS-CoV kit (Altona Diagnostics, Hamburg, Germany). For each PCR reaction, 5 µL of master A was mixed with 10 µL of master B, and 1 µL of internal control. Then, 15 µL of the master mix was added to 10 µL of the extracted RNA in a PCR tube. The reverse transcription of RNA into cDNA and the amplification for the detection of target genes *UpE* and *Orf1a* of MERS-CoV was performed using one step RT-PCR program and the Rotor-Gene Q system (Qiagen, Santa Clarita, CA, USA) according to the manufacturers’ instructions. The RT-PCR program used was 55 °C for 20 min, 92 °C for 2 min, followed by 95 °C for 15 s, 58 °C for 45 s, and 72 °C for 15 s for 45 cycles.

### 2.3. Statistical Analysis

Data were statistically analyzed using the GraphPad Prism 5 software. Statistical analysis between groups was performed using ANOVA one-way analysis of variance. Analysis between two groups was performed using unpaired t-tests. The risk ratio for mortality was determined using the Fisher’s exact test. A two-tailed *p* ≤ 0.05 was considered statistically significant.

## 3. Results

A total of 46 MERS-CoV-infected patients and 52 healthy controls were assessed. Among MERS-CoV patients, 27 (58.7%) patients were symptomatic, while 19 (41.3%) were asymptomatic. Fever was the most commonly observed manifestation, exhibited in 19 (41.3%) patients, followed by cough in 10 (21.7%) patients. Diabetes mellitus was the most frequent comorbidity, observed in 8 (17.4%) patients, followed by hypertension in 4 (8.7%) patients (Table 2). Out of 46 MERS-CoV-infected patients, 38 (82.6%) patients recovered from the infection, whereas 8 (17.4%) patients died. The recovered patients were 18 males and 20 females and their mean age was 47.42 ± 16.5 years. Moreover, 19 (50%) patients were asymptomatic and 19 (50%) patients had symptoms. Among the patients with fatal outcome, there were 4 (50%) males and 4 (50%) females and their mean age was 67.5 ± 13.6 years. All the fatal patients had symptoms and the most frequent symptoms among the patients with fatal outcome were fever, pneumonia, and shortness of breath (SOB), which was observed in 5 (62.5%) patients (Table 2).

**Figure 1:**
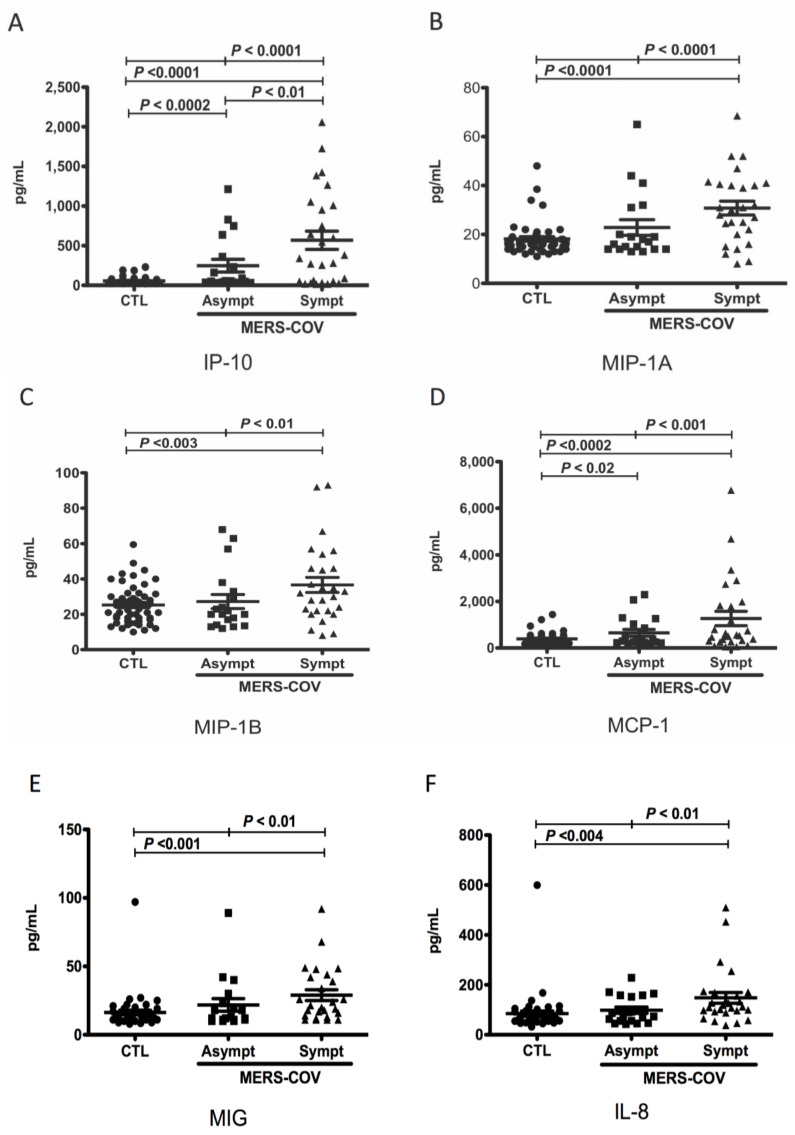

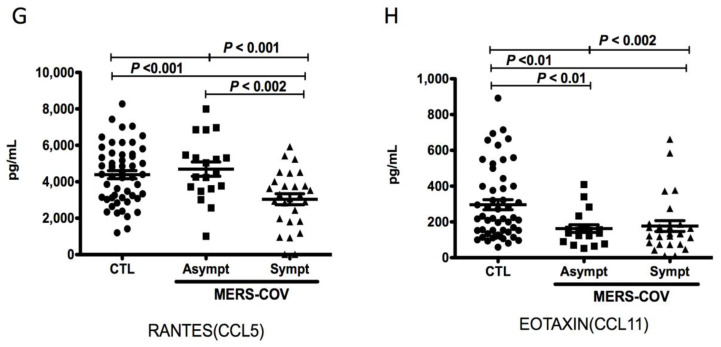
**Levels of human chemokines in study groups.** Plasma samples were collected from MERS-CoV-infected patients, symptomatic [Sympt (▲), n = 27]; asymptomatic [Asympt (■), n = 19], and healthy controls [CTL (●), n = 52)]. The levels of chemokines were measured using a customized Luminex human chemokine magnetic multiplex panel with the Luminex MAGPIX system. Plasma levels of IP-10 (**A**), MIP-1A (**B**), MIP-1B (**C**), MCP-1 (**D**), MIG (**E**), IL-8 (**F**), RANTES (**G**), and eotaxin (**H**) were plotted along with the standard error of the mean.

**Figure 2:**
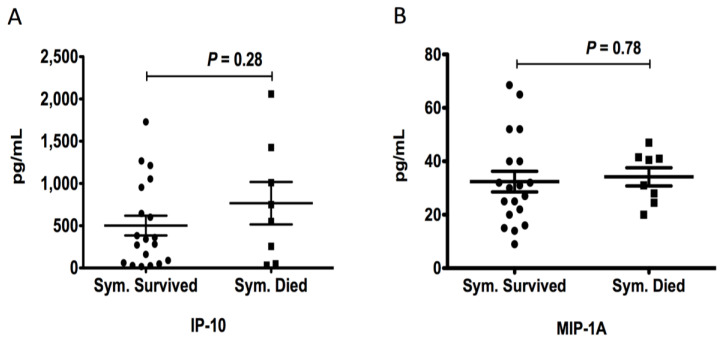

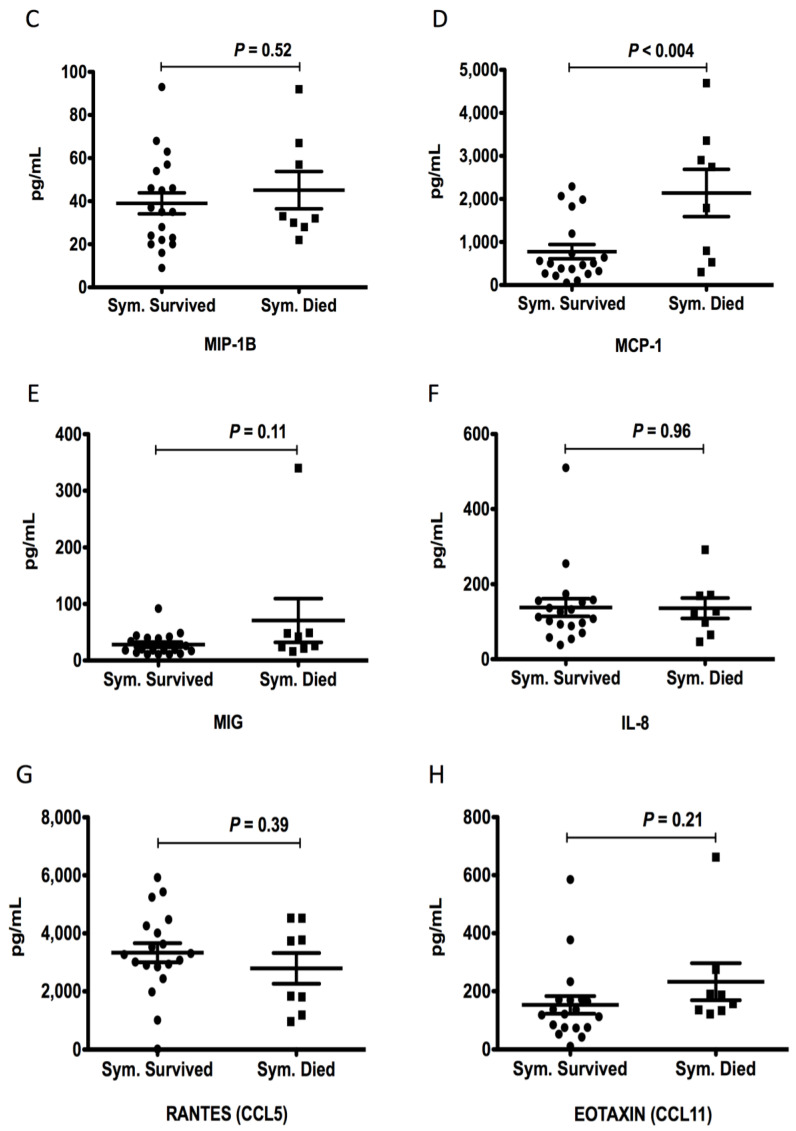
Comparison of levels of chemokines between recovered and deceased symptomatic MERS-CoV-infected patients. Plasma samples were collected from recovered symptomatic MERS-CoV-infected patients [Sym. Survived (●), n = 19] and deceased symptomatic patients [Sym. Died (■),n = 8]. The levels of chemokines were measured using a customized Luminex human chemokine magnetic multiplex panel with the Luminex MAGPIX system. Plasma levels of IP-10 (**A**), MIP-1A (**B**), MIP-1B (**C**), MCP-1 (**D**), MIG (**E**), IL-8 (**F**), RANTES (**G**), and eotaxin (**H**) were plotted along with the standard error of the mean.

Comparative analysis of the levels of chemokines between MERS-CoV-infected patients and controls revealed that the mean plasma levels of IP-10 (568.5 ± 114.7 pg/mL vs. 55.19 ± 5.85 pg/mL; *p* < 0.0001), MIP-1A (30.78 ± 2.81 pg/mL vs. 18.16 ± 0.91 pg/mL; *p* < 0.0001), MIP-1B (36.63 ± 4.25 pg/mL vs. 25.26 ± 1.51 pg/mL; *p* < 0.003), MCP-1 (1267 ± 309.5 pg/mL vs. 390.0 ± 35.51 pg/mL; *p* < 0.0002), MIG (28.96 ± 3.93 pg/mL vs. 16.29 ± 1.69 pg/mL; *p* < 0.001), and IL-8 (147.9 ± 21.57 pg/mL vs. 84.63 ± 10.62 pg/mL; *p* < 0.004) in symptomatic MERS-CoV-infected patients were significantly higher than those in healthy controls (Figure 1A–F). Moreover, we found that the mean plasma levels of IP-10 (247.6 ± 80.09 pg/mL vs. 55.19 ± 5.85 pg/mL; *p* < 0.0002) and MCP-1 (650.7 ± 149 pg/mL vs. 390 ± 35.51 pg/mL; *p* < 0.02) were also significantly higher in asymptomatic MERS-CoV-infected patients compared to controls (Figure 1A). However, we did not observe any difference in the plasma levels of MIP-1A (22.84 ± 3.19 pg/mL vs. 18.16 ± 0.92 pg/mL; *p* = 0.06), MIP-1B (27.24 ± 3.97 vs. 25.26 ± 1.51 pg/mL; *p* = 0.57), MIG (21.78 ± 4.60 pg/mL vs. 16.29 ± 1.69 pg/mL; *p* = 0.17), and IL-8 (98.32 ± 12.74 pg/mL vs. 84.63 ± 10.62 pg/mL; *p* = 0.48) between asymptomatic MERS-CoV-infected patients and controls (Figure 1B–F). In contrast, the mean plasma levels of RANTES (3039 ± 301.0 pg/mL vs. 4390 ± 223 pg/mL; *p* < 0.001) and eotaxin (176.9 ± 30.20 pg/mL vs. 296.2 ± 28.11 pg/mL; *p* < 0.01) were significantly lower in symptomatic MERS-CoV-infected patients compared to healthy controls (Figure 1G,H). The mean plasma levels of eotaxin were also significantly lower in asymptomatic MERS-CoV-infected patients compared to controls (162.7 ± 21.60 pg/mL vs. 296.2 ± 28.11 pg/mL; *p* < 0.01) (Figure 1H). The plasma level of MCP-1 was significantly higher in deceased symptomatic MERS-CoV-infected patients compared to recovered symptomatic patients (2139 ± 548.2 vs. 776.5 ± 165.3 pg/mL; *p* < 0.004) (Figure 2D). No differences in the plasma levels of IP-10 (766.4 ± 250.9 pg/mL vs. 501.6 ± 116.8 pg/mL; *p* = 0.28), MIP-1A (34.19 ± 3.4 pg/mL vs. 32.39 ± 3.87 pg/mL; *p* = 0.78), MIP-1B (45.13 ± 8.65 pg/mL vs. 39.00 ± 4.86 pg/mL; *p* = 0.52), MIG (70.81 ± 38.71 pg/mL vs. 28.13 ± 4.57 pg/mL; *p* = 0.11), IL-8 (136.1 ± 27.22 pg/mL vs. 138.0 ± 23.67 pg/mL; *p* = 0.96), RANTES (2792 ± 529.4 pg/mL vs. 3332 ± 330.2 pg/mL; *p* = 0.39), and eotaxin (232.6 ± 63.74 pg/mL vs. 153 ± 30.34 pg/mL; *p* = 0.21) were detected between deceased symptomatic MERS-CoV-infected patients and recovered symptomatic patients (Figure 2A–C,E–H). Among all the chemokines assessed, MCP-1 was the only chemokine associated with a higher risk of mortality [4.4 (95% CI (1.64–11.83); *p* < 0.017)] (Table 3).

## 4. Discussion

The mean plasma levels of IP-10, MIG, MCP-1, MIP-1A, MIP-1B, and IL-8 were higher among symptomatic MERS-CoV-infected patients compared to asymptomatic MERS-CoV-infected patients and healthy controls. Interestingly, compared with healthy controls, only the levels of IP-10 and MCP-1 were higher in asymptomatic MERS-CoV-infected patients. Members of cytokine and chemokine families IL-6, IL-8, and IP-10 are known to play a critical role in SARS-CoV and MERS-CoV infections [22]. In particular, among the chemokines, IP-10 and MCP-1 have been implicated in extensive pulmonary inflammation in SARS-CoV and MERS-CoV infections [23]. Symptomatic patients with COVID-19 requiring admission to the intensive care unit (ICU) were shown to have elevated levels of IP-10, MCP-1, and TNF-α compared with patients with less severe symptoms not requiring ICU admission [24]. Consistent with these observations, we found that the elevated levels of MCP-1 among symptomatic MERS-CoV-infected patients were associated with significantly higher risk of a fatal outcome. Moreover, in patients with COVID-19, a positive correlation observed among IL-6/IL-8, IP-10/IL-6, and IP-10/IL-8 has been linked with a poor prognosis [25]. Collectively, these observations indicate an association of the elevated levels of these cytokines and chemokines with the severity of the disease among patients with MERS-CoV, SARS-CoV, and SARS-CoV-2 infections.

Various chemokines have been implicated in the pathogenesis of SARS-CoV infections [26]. In vitro infection of monocyte-derived macrophages (MDMs) with MERS-CoV induced significantly higher concentrations of immune cell recruiting chemokines, such as IP-10, MCP-1, MIP-1α, IL-8, and RANTES compared with SARS-CoV infection of MDMs [11]. In addition, increased numbers of lung tissue-infiltrating neutrophils and macrophages were characteristically reported among patients with rapidly progressing pneumonia due to MERS-CoV infection [27]. These increased numbers of infiltrating immune cells might trigger severe inflammation and tissue damage in lung tissues, resulting in rapidly progressing pneumonia, a characteristic feature of MERS-CoV infection. The recruitment of immune cells in lungs was also confirmed by the presence of high numbers of macrophages and neutrophils in the bronchoalveolar fluids from MERS-CoV-infected patients [28]. It is highly likely that elevated levels of chemokines observed in the present study by promoting leukocyte infiltration in the tissues contributed to the manifestation of symptoms in this group.

The plasma concentrations of the C-C chemokine family members, RANTES (CCL5) and eotaxin (CCL11), were lower in symptomatic MERS-CoV-infected patients compared to healthy controls. Higher blood levels of RANTES and IL-8 have been reported to exhibit a positive correlation with the development of adult respiratory syndrome and mortality rate among patients with MERS-CoV infection [29]. The same study also reported a positive correlation between the levels of RANTES and IL-8 with lung complement anaphylotoxins C3a and C5a. Based on these observations, an elevation in the levels of C3a, C5a, IL-8, and RANTES in the lungs of MERS-CoV-infected patients was not only implicated in an increased risk of development of ARDS, but was also considered as a predictor for a fatal outcome. Eotaxins regulate eosinophil trafficking both during inflammation and homeostatic conditions [30]. Increased expression of eotaxin-1 and its receptor CCR3 has been observed in bleomycin-induced lung injury. The CCR3 receptor is also expressed on eosinophils and neutrophils, leading to increased infiltrations of these granulocytes in the lungs [31]. In contrast to these observations, there is evidence that RANTES plays a protective role in SARS-CoV-2 infection; accordingly, patients with mild COVID-19 disease were reported to have higher levels of RANTES [32]. Among patients with COVID-19, higher macrophage and monocyte infiltration in lungs was a characteristic feature compared to multinucleated giant cells, eosinophils, neutrophils and lymphocytes [33]. It is therefore possible that low eosinophil infiltration in lungs of COVID-19 patients could be due to lower expression of eotaxin. The low concentrations of RANTES and eotaxin observed in the current study among patients infected with MERS-CoV need to be investigated in larger scale studies for better elucidation of the roles of these chemokines in MERS-CoV infection.

In the current study, the gender disparity was evident among healthy controls and MERS-CoV-infected patients and was the main limitation. However, previous reports have shown no differences in the chemokine levels between healthy male and female populations [34,35]. With regards to the MERS-CoV-infected patients, the disparity may have resulted due to the grouping of patients on the basis of symptoms as the blood levels of proinflammatory mediators have been linked with the severity of disease in MERS-CoV and COVID-19 infections.

## 5. Conclusions

Elevated plasma levels of immune cell recruiting cytokines/chemokines in symptomatic MERS-CoV-infected patients contribute to the chemoattractant cascade. This might lead to massive infiltration of immune cells in the lower respiratory tract, resulting in severe pneumonia and respiratory dysfunction in patients infected with MERS-CoV. The correlation of the elevated levels of MCP-1 with a fatal outcome requires further investigations before ascertaining this chemokine as a marker of disease activity. The higher plasma levels of chemokines/cytokines among symptomatic MERS-CoV-infected patients compared with asymptomatic patients suggests that the clinical severity of MERS-CoV infection might be associated with the increased plasma concentrations of chemokines/cytokines.

## Figures and Tables

**Table 1:** Demographic details of the study groups.

Study Groups	Age (Mean ± SD) [Range]	Gender Total # (M/F)
Healthy controls	38.1 ± 13.7 [19–79] years	52 (51/1)
Asymptomatic MERS-CoV patients	44.5 ± 14.8 [19–67] years	19 (3/16)
Symptomatic MERS-CoV patients	55.4 ± 18.4 [25–93] years	27 (19/8)
Symptomatic survived	50.4 ± 18 [25–93] years	19 (15/4)
Symptomatic died	67.5 ± 13.6 [50–87] years	8 (4/4)

**Table 2:** Clinical findings of MERS-CoV positive patients.

Gender	Age	Clinical Presentation and Comorbidities	Outcome
F	25	Runny nose	Recovered
M	64	Fever	Recovered
M	61	Fever, Malaise	Recovered
F	36	Sore throat	Recovered
M	66	Fever, Disorientation	Recovered
F	69	Pneumonia	Fatal
F	52	Fever, Pneumonia	Fatal
F	75	Pneumonia	Fatal
F	87	Malaise, Body aches	Fatal
M	45	Fever, Nausea, vomiting	Recovered
M	83	Cough, Fever, Fatigue, Chest pain	Fatal
F	31	SOB, Asthma	Recovered
M	61	Fever, Chills, Rigors, DM	Fatal
M	44	Cough, Fever, DM	Recovered
M	40	SOB, Cough, Fever	Recovered
M	53	Vomiting, Nausea, SOB, Cough with sputum, Fever, DM, HTN, HF	Recovered
M	53	Vomiting, Nausea, Cough with sputum, Fever, HTN	Recovered
M	32	Cough, Fever	Recovered
M	75	SOB, Cough with sputum, DM, HTN, CAD	Recovered
F	66	Cough, Fever, DM	Recovered
M	93	Vomiting, Diarrhea, Fever, Malaise	Recovered
M	41	Fever	Recovered
M	32	Diarrhea, SOB, Fever, Malaise	Recovered
M	35	Cough, Fever	Recovered
M	63	SOB, DM, HTN, Asthma	Fatal
M	50	SOB, Cough, Fever, DM	Fatal
M	65	Fever, DM	Recovered
M	19	Asymptomatic	Recovered
F	38	Asymptomatic	Recovered
F	35	Asymptomatic	Recovered
F	41	Asymptomatic	Recovered
F	58	Asymptomatic	Recovered
F	64	Asymptomatic	Recovered
F	54	Asymptomatic	Recovered
F	60	Asymptomatic	Recovered
F	62	Asymptomatic	Recovered
F	51	Asymptomatic	Recovered
F	32	Asymptomatic	Recovered
F	59	Asymptomatic	Recovered
F	34	Asymptomatic	Recovered
F	34	Asymptomatic	Recovered
F	21	Asymptomatic	Recovered
F	29	Asymptomatic	Recovered
F	42	Asymptomatic	Recovered
M	45	Asymptomatic	Recovered
M	67	Asymptomatic	Recovered

SOB: shortness of breath; DM: diabetes mellitus; HTN: hypertension; HF: heart failure; CAD: coronary artery disease.

**Table 3:** The risk ratio of mortality for all cytokines tested.

Chemokine	Risk Ratio	95% Confidence Interval	*p* Value
IP-10	1.42	0.44–4.59	0.66
MIP-1A	2.0	0.64–6.20	0.37
MIP-1B	1.02	0.31–3.38	1.00
MCP-1	4.4	1.64–11.83	0.017 *
MIG	1.78	0.39–8.18	1.00
IL-8	1.02	0.31–3.38	1.00
RANTES	0.42	0.14–1.28	0.18
Eotaxin	1.47	0.41–5.23	0.61

* Statistically significant.

## Data Availability

Data sharing not applicable. No new data were created or analyzed in this study. Data sharing is not applicable to this article.
